# Experimental Study on the Role of Bond Elasticity and Wafer Toughness in Back Grinding of Single-Crystal Wafers

**DOI:** 10.3390/ma18214890

**Published:** 2025-10-25

**Authors:** Joong-Cheul Yun, Dae-Soon Lim

**Affiliations:** 1 Department of Materials Science and Engineering, Korea University, Seoul 02841, Republic of Korea; yunjohn@korea.ac.kr; 2 Research Center, EHWA DIAMOND, Osan-si 18145, Republic of Korea

**Keywords:** semiconductor wafer, grinding, fracture toughness, bond elastic modulus, diamond protrusion

## Abstract

Grinding semiconductor wafers with high hardness, such as SiC, remains a significant challenge due to the need to maximize material removal rates while minimizing subsurface damage. In the back-grinding process, two key parameters—the elastic modulus (Eb) of the grinding wheel bond and the fracture toughness (K_IC_) of the wafer—play a critical role in governing the behavior of diamond and the extent of wafer damage. This study systematically investigated the effect of Eb and K_IC_ on diamond protrusion height (h_p_), surface roughness (Ra), grinding forces, and the morphology of generated debris. The study encompassed four wafer types—Si, GaP, sapphire, and ground SiC—using five Back-Grinding Wheels (BGWs), with Eb ranging from 95.24 to 131.38 GPa. A log–linear empirical relationship linking h_p_ to Eb and K_IC_ was derived and experimentally verified, demonstrating high predictive accuracy across all wafer–wheel combinations. Surface roughness (Ra) was measured in the range of 0.486–1.118 μm, debris size ranged from 1.41 to 14.74 μm, and the material removal rate, expressed as a thickness rate, varied from 555 to 1546 μm/h (equivalent to 75−209 mm^3^/min using an effective processed area of 81.07 cm^2^;). For SiC, increasing the bond modulus from 95.24 to 131.38 GPa raised the average h_p_ from 9.0 to 1.2 μm; the removal rate peaked at 122.07 GPa, where subsurface damage (SSD) was minimized, defining a practical grindability window. These findings offer practical guidance for selecting grinding wheel bond compositions and configuring process parameters. In particular, applying a higher Eb is recommended for harder wafers to ensure sufficient diamond protrusion, while an appropriate dressing must be employed to prevent adverse effects from excessive stiffness. By balancing removal rate, surface quality, and subsurface damage constraints, the results support industrial process development. Furthermore, the protrusion model proposed in this study serves as a valuable framework for optimizing bond design and grinding conditions for both current and next-generation semiconductor wafers.

## 1. Introduction

In recent years, the demand for semiconductor wafers—including silicon carbide (SiC), sapphire, gallium phosphide (GaP), and silicon (Si)—has grown significantly, driven by advancements in power electronics, optoelectronic devices, and microelectromechanical systems (MEMSs). Each material possesses distinct mechanical and functional properties tailored to specific device applications. For example, SiC wafers offer exceptional electrical and thermal characteristics, such as a high dielectric breakdown field (~3 MV/cm) and thermal conductivity (~490 W/m·K), rendering them essential for high-power and high-temperature applications in electric vehicles and renewable energy systems [1,2]. Sapphire, noted for its outstanding optical transparency and mechanical robustness, is widely utilized as a substrate in the fabrication of light-emitting diodes (LEDs) and laser diodes [3,4]. GaP and other III–V compound semiconductors are extensively employed in high-efficiency optoelectronic and photonic devices due to their direct or pseudo-direct bandgap properties [5,6]. Silicon continues to dominate the CMOS and logic device sectors, owing to its superior crystalline quality and well-established manufacturing infrastructure [7].

As the semiconductor industry transitions toward larger-diameter and higher-hardness wafers, mechanical processing—particularly precision grinding—has become increasingly demanding. The exceptional hardness and brittleness of wide-bandgap semiconductors such as SiC and sapphire result in accelerated tool wear, elevated grinding forces, and pronounced surface and subsurface damage during wafer thinning. To address these challenges, diamond grinding wheels with diverse bond systems are utilized to achieve an optimal balance between material removal rate, surface integrity, and tool longevity [8,9]. Accordingly, a comprehensive understanding of the interrelationship between elastic bond modulus, abrasive grain protrusion, and wafer fracture toughness is essential for enhancing grinding performance across various semiconductor substrates.

Various grinding and polishing techniques have been studied to reduce subsurface damage and improve the surface quality of semiconductor wafers. Li et al. [10] applied shear rheological polishing to silicon wafers, while Wang et al. [11] investigated fixed-abrasive lapping on sapphire wafers under various process conditions and studied eco-friendly chemical mechanical polishing (CMP) using slurry to enhance surface quality. For SiC wafers, Gong et al. [12] reported CMP using optimized slurry, and Wang et al. [13] demonstrated photo-assisted CMP (PCMP). In addition, Wu et al. [14] employed friction-induced chemical polishing using a pure iron plate. To further reduce surface roughness and subsurface damage, Luo et al. [15] proposed the use of a soft–hard composite grinding wheel, and Ye et al. [16] introduced ultrasonic vibration-assisted grinding.

While numerous prior studies have demonstrated improvements in wafer surface quality by primarily targeting the reduction in surface roughness, these investigations were generally conducted under extremely low material removal rate (MRR) conditions—typically less than a few micrometers per hour. Such limitations hinder their practical applicability in high-throughput semiconductor manufacturing environments. In this context, the back-grinding process plays a pivotal role by rapidly thinning wafers from several hundred micrometers to several tens of micrometers. Attaining a high MRR is therefore crucial for enhancing productivity and minimizing manufacturing costs [17,18].

This study aims to elucidate the grinding mechanism that enables efficient material removal in the high-MRR regime—a domain that has received limited attention in prior research. Specifically, it quantitatively investigates the relationship between diamond protrusion height (h_p_) and elastic bond modulus (Eb), both of which critically influence high-MRR performance. While previous studies have primarily focused on single-material wafers, such as Si or SiC, they often overlook the impact of wafer material properties on grinding behavior. In contrast, the present work systematically compares the back-grinding characteristics of four distinct wafer types—Si, GaP, sapphire, and SiC—each exhibiting unique mechanical properties, including Eb and fracture toughness (K_IC_). By clarifying how these material attributes affect h_p_ and MRR dynamics, this research provides foundational insights for developing optimized process conditions tailored to diverse semiconductor substrates in practical manufacturing environments.

Previous studies have independently linked abrasive protrusion to factors such as dressing conditions, bond stiffness, or wafer brittleness. However, to the best of our knowledge, no concise predictive model has been proposed that concurrently incorporates both Eb and K_IC_ to account for cross-material variations in protrusion behavior. In this study, a log–linear formulation is introduced that integrates these parameters and demonstrates applicability across four types of single-crystal wafers using a unified parameter set. This approach facilitates a grinding wheel design that reflects material-specific mechanical properties, thereby enhancing process optimization.

The proposed model establishes an experimental relationship between Eb and K_IC_ to estimate h_p_. This enables the rapid formulation of initial processes that achieve target MRR and surface roughness with minimal reliance on the design of experiments (DOE) while accounting for material-specific characteristics. Operating at an optimal Eb reduces grinding load, thereby lowering energy consumption, extending wheel life, and decreasing dressing frequency—ultimately minimizing consumable usage and equipment downtime. By mapping volumetric MRR to thickness removal rate (μm/h), process output can be aligned with takt time, and stable control of subsurface damage (SSD) and chipping helps reduce reworking and scrapping. This workflow facilitates efficient process transfer across diverse wafer materials—including Si, GaP, sapphire, and SiC—and is scalable to larger diameters through area-based conversion, delivering enhanced throughput at reduced overall cost. However, the model exhibits optimal performance for calibrated bond types and grit conditions and may require recalibration in extreme operating environments or when applied to ductile or polycrystalline substrates.

Therefore, this study presents a high-throughput, multi-material-oriented approach to back grinding, offering significant academic and industrial relevance beyond conventional research.

## 2. Materials and Experimental Methods

### 2.1. Preparation of Evaluation Wafers

In this study, four types of wafers with different material properties—Si (4-inch, Shin-Etsu, Tokyo, Japan), GaP (4-inch, Shin-Etsu, Tokyo, Japan), sapphire (4-inch, Iljin Diamond, Seoul, Republic of Korea), and 4H-SiC (4-inch, SICC, Jinan, China)—were evaluated to investigate grinding mechanisms based on their material characteristics. The grinding characteristics of wafers were evaluated by selecting surfaces susceptible to brittle fracture based on variations in fracture toughness. For silicon wafers, the (100) surface exhibits higher susceptibility to crack initiation and lateral fracture compared to the (111) surface, resulting in better machinability [19]. Similarly, the (110) surface was selected for GaP wafers [20] and the C-plane (0001) surface for sapphire wafers, as they are more prone to brittle fracture during processing [21]. For SiC wafers, between the Si-face (0001) and the C-face (000-1), the Si-face (0001) was chosen for evaluation due to its higher tendency for brittle fracture [22].

The mechanical properties of these wafers were measured using a micro-Vickers tester to determine hardness and Eb. K_IC_ was assessed using the nanoindentation technique proposed by Lawn and Evans [23]. Crack lengths induced by the indenter were measured, and the K_IC_ of each wafer was calculated using Equation (1), as suggested by the authors. The measured values of hardness and Eb for each wafer are presented in Table 1.

(1) $$K_{IC}=\alpha \cdot {\left(\frac{E}{H}\right)}^{\frac{1}{2}}\cdot \left(\frac{P}{c^{3/2}}\right)$$

where P is the indentation load, E is the elastic modulus, H is the hardness, c is the total length from the center of the indent to the end of the crack, and α is a constant (0.016) related to the crack morphology and the shape of the indenter used [24].

### 2.2. Fabrication of the Back-Grinding Wheel

To vary the elastic modulus of the bond supporting the diamond in this experiment, by incorporating 10–50 wt% of Co into the Cu-Sn matrix bond, Eb was controlled to fabricate a Back-Grinding Wheel (BGW) [25,26]. The bond materials used for wheel fabrication were bronze metal powder (SF-BR8020, Nippon Atomized Metal Powders Co., Japan) and cobalt powder (EF, Umicore, Belgium), which were mixed with diamond powder (MBG-660 #325/400, Hyperion Materials & Technologies, USA) to achieve a relative density of 90%. The design parameters of the wheel (diamond and metal powder composition), along with the relative density and Eb measured using the Archimedes method and flexural strength evaluation, are presented in Table 2.

To isolate the effect of the bond’s elastic modulus on protrusion height and grinding responses, abrasive parameters were held constant across all wheels. A median diamond size of 51.2 µm (≈D54) was chosen because, under our dressing condition, it yields a stable protrusion h_p_ = 10−15 µm suited to brittle single-crystal wafers (Si, GaP, sapphire, SiC) under the selected process parameters. The diamond content was fixed at 12.5 vol% to balance active-grain density, bond integrity, and coolant access; pilot tests showed that higher contents (>~20 vol%) increased pull-out and bond micro-fracture, whereas lower contents (<~8 vol%) reduced MRR and raised local forces.

### 2.3. Grinding Wafer by Back-Grinding Equipment

**Figure 1:**
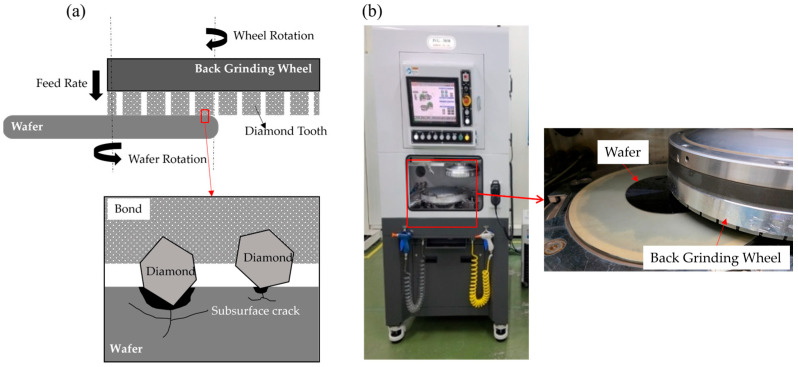
(**a**) Wafer back-grinding mechanism and (**b**) photograph of the back-grinding equipment and close-up of wafer grinding interface.

Figure 1a illustrates a schematic diagram of the back-grinding process. On the surface of the BGW, diamonds protrude from the diamond teeth. During grinding, diamonds remove material from the wafer surface as they traverse it, driven by the relative rotation between the grinding wheel and the wafer. Depending on h_p_, the diamonds penetrate the wafer, initiating vertical and horizontal cracks from the contact point. These cracks propagate, leading to fracture in wafers with low tensile strength [27].

As shown in Figure 1b, the wafer grinding equipment used in this study is the IVG-3030 model from INSEMITEC, Korea, which is commonly employed in the wafer thinning process for semiconductor manufacturing. A key feature of this equipment is its ability to control the spindle rotation speed from 500 to 3000 RPM (Revolutions Per Minute) via a position sensor mounted on the rotating axis. It also allows for precision cutting depths of up to 1 μm and is equipped with a built-in tool dynamometer capable of measuring the grinding force during operation.

In this experiment, the BGW was rotated clockwise at a peripheral speed of 23.5 m/s, while the wafer was rotated in the opposite direction at 1.6 m/s. The feed rate was set to 0.5 μm/s. To evaluate the MRR, a total of 200 μm of wafer thickness was removed during the grinding process. Back grinding was performed on four types of wafers using five different BGWs. The load generated during processing was measured using an integrated tool dynamometer. Post-grinding evaluations included analysis of the diamond condition in the BGWs and measurement of wafer surface roughness using an optical 3D surface profiler (Wyko NT3300, Veeco, USA). The size of the wafer debris generated during the grinding process was measured by collecting the slurry produced after grinding, drying it in an oven (80 °C for 3 h), and obtaining images using a field-emission scanning electron microscope (FE-SEM, Axia ChemiSEM, Thermo Fisher Scientific, USA). The debris size of Si, GaP, sapphire, and SiC wafers was then analyzed using the image processing program (Image Pro Plus, Media Cybernetics, USA).

### 2.4. Measurement of Diamond Protrusion Height (h_p_)

**Figure 2:**
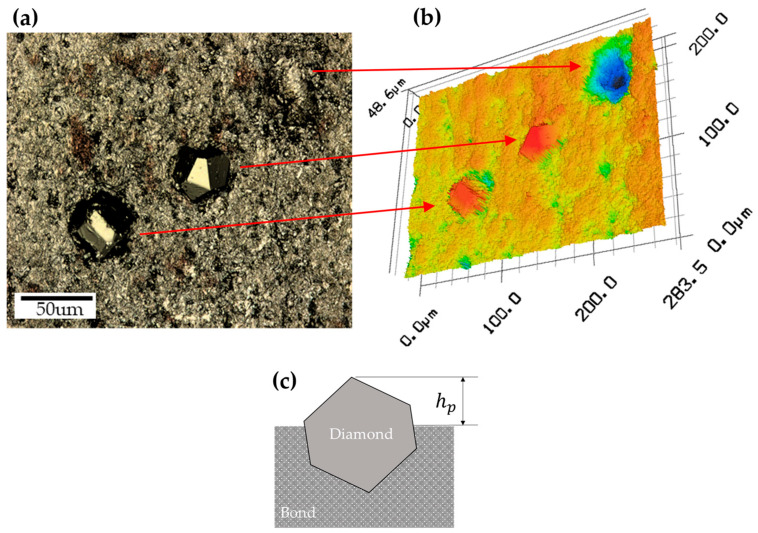
Measurement method for diamond protrusion height on grinding wheel surface: (**a**) microscopic image; (**b**) profile image; and (**c**) definition of diamond protrusion height.

h_p_ was measured using a confocal microscope (VK-910K, KEYENCE, Japan). Figure 2a shows the shape of the diamond captured using an optical microscope at 50× magnification. Figure 2b presents an image of the measured diamond, and as illustrated in Figure 2c, h_p_ was determined by measuring the diamond that protruded the most from the grinded surface of the diamond tooth [28,29]. A BGW is fabricated by bonding 42 diamond teeth onto the wheel. In this study, the maximum protrusion height of the diamond in each tooth is defined as h_p_.

## 3. Results and Discussion

### 3.1. Diamond Protrusion Height with Elastic Bond Modulus

**Figure 3:**
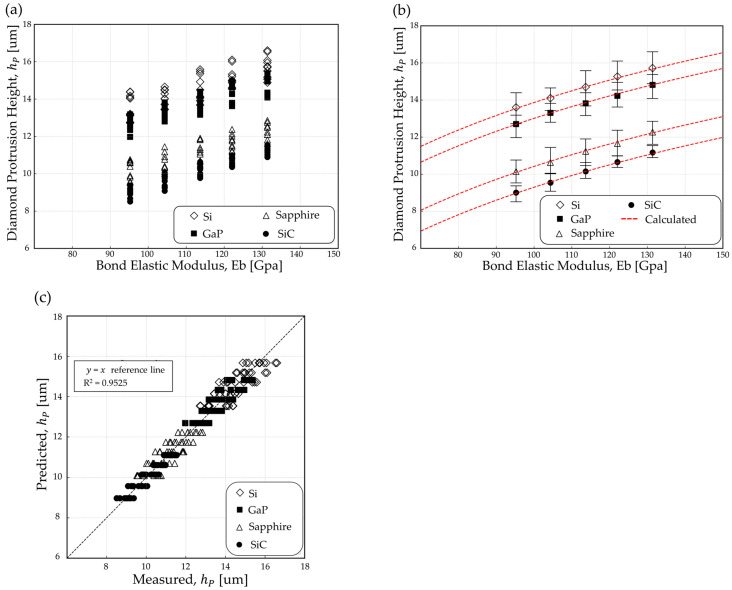
Measured and calculated values of diamond protrusion height with elastic bond modulus after back grinding of semiconductor wafers: (**a**) raw scatter data and (**b**) fitted regression line for the average data (red dotted line); (**c**) a parity plot with the identity line (y = x) as the reference.

From the evaluation of the grinding performance using wafers with different material properties and various BGWs, the measured h_p_ values are presented in Figure 3.

Figure 3a presents the raw data, while Figure 3b overlays the calculated fitted curve (red dotted line) along with the corresponding equation and goodness-of-fit metrics such as R^2^.

During the grinding process, the h_p_ of diamonds can be influenced by displacement caused by the elastic modulus of the bond material. This study confirmed that as the elastic modulus of the bond increases, h_p_ tends to increase.

**Figure 4:**
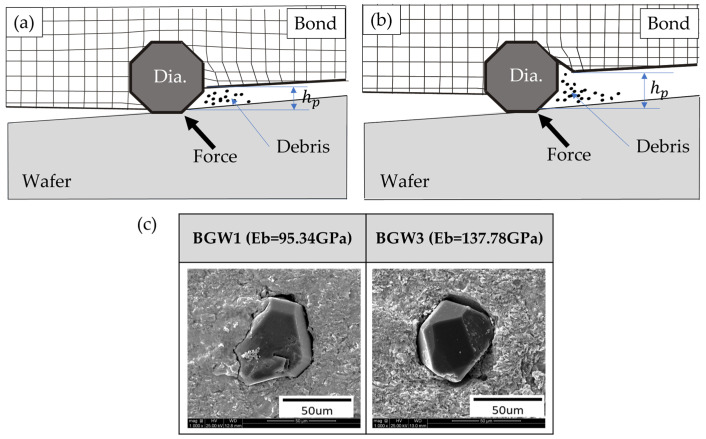
Schematic of the diamond protrusion process in BGWs: (**a**) low elastic bond modulus; (**b**) high elastic bond modulus; and (**c**) image of a protruding diamond.

As shown in Figure 4a, BGWs with a lower Eb tend to deform more easily under grinding forces. This deformation causes the diamond to be pressed deeper into the bond, thereby reducing its h_p_. For this reason, it can be observed in Figure 4c that the diamonds processed with BGW1, which has a low Eb, are embedded into the bond.

Conversely, Figure 4b demonstrates that BGWs with higher Eb exhibit minimal deflection, which helps maintain a greater h_p_ and facilitates more effective transmission of grinding forces. It is shown in Figure 4c that grinding with BGW5, which has a high Eb, leads to interfacial separation between the bond and the diamond due to the repetitive forces generated during grinding.

During continuous grinding cycles, localized plastic deformation occurs at the diamond–bond interface, leading to the formation of voids that trap debris. As debris accumulates, bond wear accelerates, resulting in a gradual increase in h_p_. This phenomenon is influenced not only by the elastic modulus of the bond but also by various material properties, such as the size and hardness of the debris and the K_IC_ of the wafer. In particular, large or hard debris intensifies bond wear, further increasing diamond protrusion during prolonged grinding [30]. In addition, even when using the same grinding wheel, the h_p_ values for sapphire and SiC wafers were lower than those for Si and GaP wafers. This is because wafers with higher K_IC_ tend to generate smaller debris during grinding, which reduces bond wear and consequently suppresses abrasive grain protrusion.

h_p_ was determined by both the Eb of the wheel bond material and the K_IC_ of the wafer. The empirical model proposed in this study reflects the influence of wafer K_IC_ on debris formation, which in turn affects the degree of diamond protrusion.

It was found that the protrusion height of diamond particles (h_p_) is influenced by the elastic modulus (Eb) of the wheel bond and the fracture toughness (K_IC_) of the wafer. To analyze the experimental results, an empirical model was developed in this study.

Specifically, an empirical–mechanistic hybrid model was introduced, based on mechanical and fracture mechanics principles, to describe how the diamond protrusion height (h_p_) is governed by the elastic modulus (Eb) of the BGW bond and the fracture toughness (K_IC_) of the wafer material.

Based on the experimental results, a predictive model was constructed using Eb [25] and the wafer K_IC_ [24,31] as variables affecting h_p_, as shown in Equation (2).

(2) $$h_{p}=b\cdot log\left(\frac{E_{b}}{E_{0}}\right)+\alpha \sqrt{\frac{K_{0}}{K_{IC}}}+C$$

h_p_ (μm) represents the h_p_ that may occur during the grinding process, Eb (GPa) denotes the elastic modulus of the BGW bond, and K_IC_ (MPa·m^0.5^) indicates the K_IC_ of the wafer. The coefficients b, α, and C were determined based on experimental data, and E_0_; = 1 GPa and K_0_; = 1 MPa·m^0.5^ were set as reference values. To avoid dimensionalizing the logarithmic and square-root terms in Equation (2), reference constants were fixed at E_0_ = 1 GPa and K_0_ = 1 MPa⋅m^0.5^ rather than using dataset-dependent geometric means. This unity anchoring keeps the predictors strictly dimensionless, avoids embedding sample-specific central tendencies, and facilitates reproduction and cross-study comparison. Mathematically, changing E_0_ only adds a constant that is absorbed by C, and changing K_0_ rescales α; predictions are invariant under such re-parameterizations.

The parameter b represents the sensitivity of h_p_ to changes in Eb. A larger b value indicates that the increase in h_p_ becomes more pronounced as Eb increases. This quantitatively describes the phenomenon in which, as the bond becomes harder, the abrasive grains are less likely to be pressed into the bond, resulting in enhanced support and greater protrusion [32]. α is a sensitivity coefficient that reflects the influence of wafer K_IC_ on the size of the generated debris. According to G. R. Anstis et al. [33], α is not merely a scaling constant, but rather an empirical correction factor that accounts for crack morphology, plastic deformation behavior during indentation, and specific experimental conditions. When the bond is extremely soft, the diamond particles are easily pressed in, resulting in minimal protrusion and deep embedding within the bond. In this case, C is defined as the reference value for the embedding depth of the particles in an extremely soft bond.

Equation (2) was formulated as a semi-empirical relation constrained by the bond mechanics and brittle fracture scaling of the wafer. The bond elasticity contribution is expressed as a natural-log transform of the bond modulus, ln(Eb/E_0_), to reflect the monotonic increase in diamond retention/protrusion with stiffness and to reduce the influence of discrete Eb levels. The wafer toughness contribution is expressed as a square-root term of the inverse toughness, $\sqrt{K_{0}/K_{IC}}$, consistent with classical crack penetration scalings in brittle materials. A constant term captures the baseline set by the fixed grit size, diamond fraction, and dressing protocol used in this study. Reference values E_0_ and K_0_ were set to the geometric means of the tested levels to avoid dimensionalizing the predictors and reduce parameter correlation.

For each (material, Eb) condition, replicate protrusion measurements (h_p_) were averaged and their variances were retained. Parameters were estimated by weighted least squares (WLS), using weights of ω = 1/Var(h_p_) to account for heteroscedasticity across cond (itions. Uncertainty was quantified by 95% confidence intervals from the estimated covariance matrix and corroborated by bootstrap resampling at the condition level. Model adequacy was assessed by residual analysis (residuals vs. fitted and predictors) and by monotonicity checks (increasing h_p_ with Eb; decreasing h_p_ with K_IC_).

Generalization was assessed using leave-one-wheel-out and leave-one-material-out validation checks, with the goodness of fit (R^2^) reported alongside the results. Several alternative formulations—including a pure logarithmic model in Eb, a power-law model in Eb, and bilinear or saturating variants—were evaluated based on cross-material performance. Equation (2) was ultimately retained as the most parsimonious model, consistently capturing the effects of bond stiffness and wafer toughness with minimal parameters. The h_p_ values predicted by Equation (2) were compared with the measured h_p_ values, and the coefficient of determination (R^2^) is presented in Figure 3c using a parity plot with the identity line (y = x) as the reference.

A multiple regression analysis (log–linear regression) was performed using Minitab Statistical Software (Release 16.2.3) to investigate the relationship between h_p_ and the parameters Eb and K_IC_ based on various experimental results [23,25]. The analysis revealed that h_p_ exhibits a linear dependence on the logarithm of Eb and the inverse of the wafer K_IC_. The coefficients b and α and constant C were determined by fitting the experimental data. The calculated and measured values are compared under the parameter conditions b = 6.6310, α = 7.0868, and C = −25.6047 with a coefficient of determination R^2^ = 0.9525 in Figure 3b.

The red dotted trends shown in Figure 3b were generated by evaluating Equation (2) with the WLS-estimated parameters at the measured Eb levels for each wafer (using its K_IC_). The fit is calibrated for our wheel design and kinematics (median diamond size ≈ 51.2 µm; diamond fraction ≈ 12.5 vol%; fixed dressing protocol; and the speeds/feeds listed). Extrapolation to highly porous or highly elastic bonds, extreme grit sizes/concentrations, markedly different dressing states, or ductile/polycrystalline substrates should be undertaken with caution. Wafer orientation was held constant in this study and may shift absolute levels without altering the reported dependencies on Eb and K_IC_.

Figure 3b shows the values predicted by Equation (2) for four wafer types—silicon (Si), gallium phosphide (GaP), sapphire, and silicon carbide (SiC). For all tested wafers, the empirical model produced h_p_ predictions that closely matched the experimental measurements, indicating that the combined effects of Eb and wafer K_IC_ govern diamond exposure. The small discrepancies observed between calculated and measured values suggest that the model can reliably estimate h_p_ across a range of grinding wheel compositions and workpiece materials.

Distinct differences were observed among the four wafer types: for a given value of Eb, the h_p_ values followed the order Si > GaP > sapphire > SiC. This ranking is consistent with the wafers’ K_IC_, measured at 0.63, 0.77, 1.67, and 2.64 MPa·m^0.5^ for Si, GaP, sapphire, and SiC, respectively, as shown in Table 1. h_p_ can be controlled by adjusting the Eb of the diamond wheel, demonstrating its applicability to the machining of various types of wafers.

### 3.2. Diamond Protrusion Height (h_p_) and Grinding Outcomes

**Figure 5:**
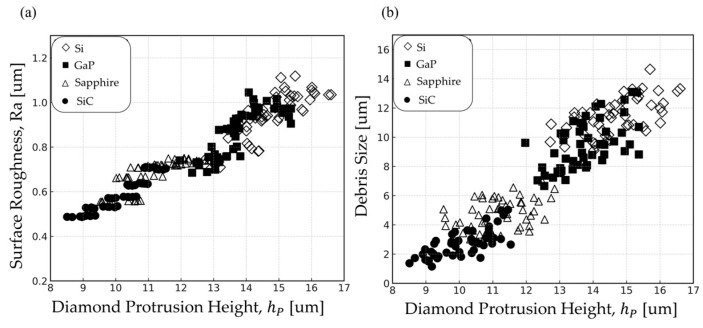
Relationship between diamond protrusion height (h_p_) and grinding outcomes: (**a**) surface roughness (Ra); (**b**) debris size for Si, GaP, sapphire, and SiC wafers.

To investigate the influence of h_p_ on wafer grinding, the measured wafer surface roughness and debris size were correlated with the h_p_ values, as shown in Figure 5.

Figure 5a presents the measured values of both h_p_ and surface roughness obtained in this study. Across all materials, Ra increased linearly with rising h_p_. Surface roughness appears to be significantly influenced by h_p_, regardless of the wafer material.

Figure 5b illustrates the variation in h_p_ with respect to debris size. As the debris size increased, the h_p_ also tended to increase, consistent with the mechanism described in Figure 4. Additionally, the debris size ranking among wafer materials follows the order: Si > GaP > sapphire > SiC. This trend is closely related to the K_IC_ of the wafers. Materials with lower K_IC_, such as Si and GaP, are more prone to crack initiation and propagation, resulting in the formation of larger debris. In contrast, materials with higher K_IC_, such as sapphire and SiC, suppress crack formation and produce finer particles. The average debris sizes were approximately 9–15 μm for Si, 7–13 μm for GaP, 3–7 μm for sapphire, and 1–5 μm for SiC.

Debris size is a key factor influencing the self-renewing capability of the BGW during the back-grinding process. This is because debris promotes bond erosion and cavity formation, effectively exposing new abrasive grains and helping to maintain the grinding edge [28]. Therefore, debris size could also be an important parameter affecting overall grinding performance in the wafer back-grinding process that should be controlled.

In addition, the debris size was analyzed based on the K_IC_ of the wafers. Evans et al. [23] reported that, in brittle materials, debris size is inversely proportional to K_IC_^2/3^. In this study, wafers with different K_IC_ values were processed, and the resulting debris sizes were measured to examine whether the debris generated during back grinding exhibits a similar trend.

**Figure 6:**
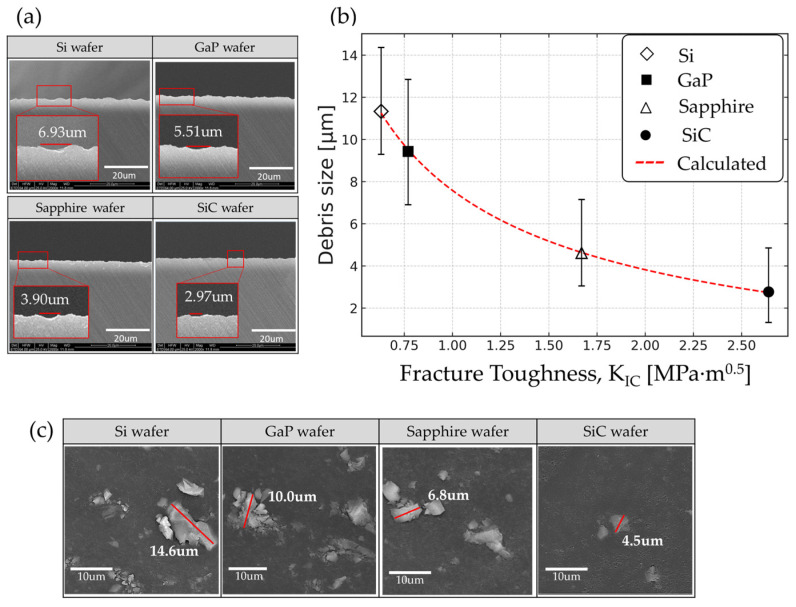
(**a**) Chipping of wafers, (**b**) relationship between fracture toughness (K_IC_) and debris size, and (**c**) SEM images of generated debris.

Surface chipping patterns for wafer grinding using the same BGW are shown in Figure 6a, and the image in Figure 6c presents the measurement of debris generated from the wafers during this process. The larger debris size relative to the on-surface chipping span arises from 3D fracture geometry and post-event material removal. In brittle grinding, subsurface lateral cracks propagate roughly parallel to the surface; the detached flakes therefore reflect the lateral crack diameter in plane, whereas the residual surface cavity records only a smaller fraction of that crack network (e.g., debris ≈ 14.6 µm vs. pit ≈ 6.93 µm). In addition, subsequent wheel passes trim the pit lips, further shrinking the apparent on-surface span, while the collected debris retains its original maximum Feret length.

For Si and GaP, which have relatively low K_IC_ values, extensive lateral chipping occurs due to greater crack propagation. In contrast, sapphire and SiC exhibit only minimal localized chipping. To quantitatively analyze the trend observed in Figure 6a, wafers with different K_IC_ values were ground using BGW1 to BGW5, and the results are plotted in Figure 6b. The red solid line represents the fitted curve calculated based on the Evans model, which is expressed as Equation (3). This equation shows strong agreement with the experimental results, yielding a coefficient of determination (R^2^) of 0.985 with fitting parameters a = 10.17 and b = –2.59.

(3) $$Debris\;Size,d=a\cdot {\left(\frac{1}{K_{IC}}\right)}^{2/3}+b$$

This trend is consistent with previous studies [23,33] which reported that materials with lower K_IC_ (i.e., more brittle) tend to propagate cracks more easily, resulting in larger debris, whereas materials with higher K_IC_ suppress crack propagation, leading to the formation of finer debris.

### 3.3. Evaluation of Grinding Wafers

**Figure 7:**
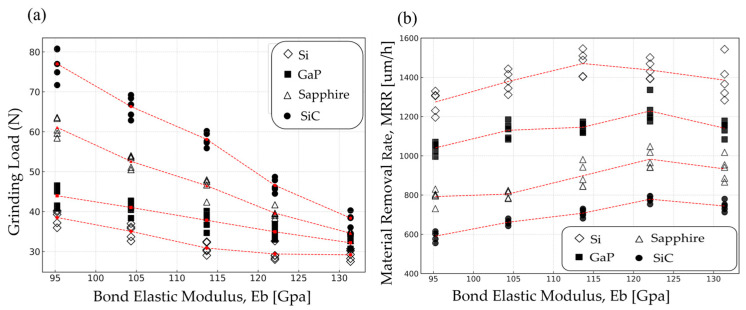
Grinding performance against elastic bond modulus: (**a**) grinding load; (**b**) material removal rate for Si, GaP, sapphire, and SiC wafers.

The grinding loads and MRRs measured during the grinding evaluation using wheels with different elastic moduli are presented in Figure 7.

Figure 7a shows the maximum grinding load generated during the back-grinding process. The Eb is a critical factor that directly affects the grinding load. When the bond is soft (i.e., low Eb), frictional interaction increases, leading to force accumulation and a higher grinding load. In contrast, a strong bond (i.e., high Eb) shifts the cutting mode toward fracture-based removal, allowing smaller fragments to separate more easily and thereby reducing the grinding load [27]. For materials such as Si and GaP, which are inherently brittle, the effect of bond strength variation is relatively minor. However, in the case of SiC, which has high mechanical strength, the influence of bond support becomes significantly more pronounced, resulting in a more noticeable reduction in grinding load.

As shown in Figure 7b, the K_IC_ of the wafer significantly influences the MRR during the grinding process. For materials with low K_IC_, such as Si and GaP, a relatively high MRR can be achieved even with a low Eb, and the MRR tends to saturate beyond a certain threshold. In contrast, materials with high K_IC_, such as sapphire and SiC, exhibit strong resistance to crack propagation, requiring a higher Eb to ensure a sufficient MRR. However, when Eb becomes excessively high, the bond itself becomes overly rigid, leading to stress concentration at the interface between the diamond and the bond. This results in accelerated bond wear due to the high grinding forces and impact from debris generated during grinding. Consequently, localized bond detachment around the diamond, micro-fracture of diamond particles, and premature diamond loss may occur. These effects shorten the lifespan of effective grinding diamonds and reduce the overall grinding efficiency, ultimately leading to a decrease in MRR [22]. Therefore, the optimal range of Eb varies depending on the K_IC_ of the wafer material. According to the results of this study, an Eb of 122.07 GPa was found to be the most suitable value for grinding SiC.

**Figure 8:**
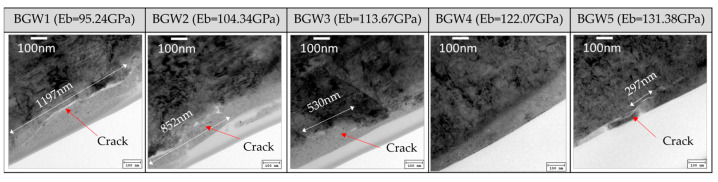
Internal cracking of SiC wafer after grinding.

Figure 8 presents the cross-sectional TEM analysis results of SiC wafers ground using BGW1 through BGW5. For BGWs with low Eb, lateral cracks were observed, which are attributed to tensile stress concentration caused by insufficient support from the diamond abrasives during grinding. The crack size decreased progressively from 1197 nm (BGW1) to 852 nm (BGW2) and 530 nm (BGW3), while no cracks were observed for BGW4. However, BGW5—with an excessively high elastic bond modulus (Eb)—exhibited minimal bond deformation, causing the grinding force to be directly transmitted to the wafer and resulting in localized cracking of 29 nm. Among the tested wheels, BGW4, with an Eb of 122.07 GPa, demonstrated the most stable grinding load conditions and achieved the highest MRR without inducing subsurface damage. Using the corresponding grinding wheel, the machining of SiC wafers showed stable grinding loads ranging from 45.92 to 49.67 N. The MRR was measured at 753.87 to 794.94 um/h, and the surface roughness remained consistent within a range of 0.627 to 0.638 um, indicating uniform grinding quality.

These findings suggest that grinding efficiency is governed by a balanced interplay among Eb, diamond protrusion, and the mechanical properties of the wafer—where K_IC_ emerges as a critical factor. Despite the inherent difficulty of grinding SiC due to its exceptional hardness and toughness, grinding wheels with a high Eb help maintain diamond protrusion and bond integrity. This enables effective material removal while preserving surface quality.

While the relationship between Eb, material toughness, and internal cracking is established here, a wheel-resolved SSD depth dataset remains outstanding. This will be addressed in follow-up studies using serial cross-sectioning (mechanical/FIB), calibrated etching, and 3D imaging to produce quantitative SSD depth maps across all wheels and materials.

## 4. Conclusions

A cross-material experimental study was conducted on Si, GaP, sapphire, and SiC using five back-grinding wheels spanning Eb = 95.24–131.38 GPa at fixed grit size and concentration. Diamond protrusion height (h_p_), surface roughness (Ra), debris size, material removal rate, and grinding load were measured.

- The proposed diamond protrusion model: The protrusion height (h_p_) increases logarithmically with Eb, while it decreases as K_IC_ increases. This relationship is well described by Equation (2). The empirical model accurately predicts h_p_ across various combinations of Eb and K_IC_, providing a valuable tool for grinding wheel design and process optimization.
- Grinding load and MRR: Increasing Eb enhances force transmission efficiency, resulting in reduced grinding loads across all wafer types. This effect is particularly pronounced in materials with high K_IC_, such as SiC. For the SiC wafer, using a BGW with an Eb of 122.07 GPa enabled stable grinding under a load of less than 50 N, achieved an MRR exceeding 740.1 um/h (100 mm^3^/min), and resulted in a surface roughness (Ra) of approximately 0.633 um.
- How to use the model: The proposed log–linear relation links Eb and K_IC_ to predict h_p_. In practice, (i) candidate bonds are selected to span a target Eb window, (ii) the wafer’s K_IC_ is inserted to predict the h_p_, and (iii) the predicted h_p_ is mapped to the allowable uncut-chip thickness/SSD to determine appropriate feed and infeed parameters. A short 3–5-point DoE then locks the bond–dressing pair that meets the MRR/roughness targets.
- Guidelines for wheel bond design and selection: Within our tested window (≈95–131 GPa), stiffer bonds systematically increase h_p_ and reduce grinding load; however, excessively high Eb can elevate interfacial stresses and accelerate bond micro-fracture/pull-out.
- (i) Low-toughness wafers (e.g., Si, GaP): Moderate Eb is prioritized to balance h_p_ and edge-chipping control.
- (ii) High-toughness wafers (e.g., sapphire, 4H-SiC): The upper Eb range is used to secure sufficient h_p_ but paired with controlled dressing to avoid overly stiff behavior.
- (iii) Grit size and concentration are fixed when isolating bond effects; dressing severity is adjusted to fine-tune h_p_ around the model’s prediction.
- Process parameter optimization: Predicted h_p_ is used to initialize feed/infeed and finishing passes: coarse removal at the h_p_-guided set-point, then a fine-grit, low-load final pass to minimize SSD/chipping. Spindle power/load, debris size, and Ra are monitored as shop-floor proxies for h_p_ to maintain stability and schedule dressing.
- Industrial impact: The workflow shortens process transfer across Si, GaP, sapphire, and SiC, raises throughput at a given roughness/SSD, and reduces cost via lower energy, fewer dressings, and longer wheel life. Applicability is strongest within the calibrated bond/grit/dressing ranges; extreme bonds, grit sizes/concentrations, or ductile/polycrystalline substrates may require re-fitting.

These findings validate the predictive model and highlight the role of Eb in maintaining diamond protrusion and minimizing surface damage. By tailoring wheel design to the K_IC_ of the wafer, it is possible to enhance material removal efficiency without compromising surface integrity, even in the machining of ultra-hard substrates like SiC.

The model was calibrated under specific bond types, a median grit size (~51 µm), a diamond fraction (~12.5 vol%), and fixed dressing/kinematic conditions; therefore, extrapolation to highly porous or elastomeric bonds, extreme grit sizes or concentrations, or markedly different thermal/mechanical environments should be approached with caution. In this study, the crystallographic orientation is held constant within each material, so the reported Eb–K_IC_ relationships are not confounded, although orientation-dependent offsets in absolute values may exist and will be quantified in future work. A wheel-resolved SSD depth dataset was not acquired; instead, lateral crack metrics and debris size were reported as informative proxies, with systematic SSD depth mapping deferred to follow-up studies (serial cross-sectioning/3D imaging). Forthcoming work will broaden validation to additional semiconductors (e.g., GaN, SiGe), wider bond/grit/dressing ranges and speed/feed windows, and other production factors (coolant chemistry/temperature, wheel wear/life, scaling to larger wafer diameters). These efforts will refine parameter estimates and, if warranted, incorporate orientation and SSD depth descriptors, further translating the framework into factory-ready designs for controlling h_p_–Ra–chip performance and SSD.

## Figures and Tables

**Table 1:** Physical properties of Si, GaP, sapphire and SiC wafer.

	Si Wafer	GaP Wafer	Sapphire Wafer	SiC Wafer
Size	81.07 cm^2^	81.07 cm^2^	81.07 cm^2^	81.07 cm^2^
Grinding surface	plane (100)	Plane (110)	c-plane (0001)	Si-Face (0001)
Hardness (Vickers)	1050 ± 113 Kg/mm^2^	1154 ± 53 Kg/mm^2^	1918 ± 128 Kg/mm^2^	3445 ± 189 Kg/mm^2^
Elastic modulus	135 ± 15 GPa	292 ± 33 GPa	347 ± 39 GPa	422 ± 48 GPa
Fracture toughness (K_IC_)	0.63 ± 0.024 MPa·m^0.5^	0.77 ± 0.048 MPa·m^0.5^	1.67 ± 0.021 MPa·m^0.5^	2.64 ± 0.019 MPa·m^0.5^

**Table 2:** Properties of BGWs.

	BGW1	BGW2	BGW3	BGW4	BGW5
Cobalt Content	10 wt%	20 wt%	30 wt%	40 wt%	50 wt%
Diamond Size	Average 51.2 um	Average 51.2 um	Average 51.2 um	Average 51.2 um	Average 51.2 um
Diamond Content	12.5 Volume%	12.5 Volume%	12.5 Volume%	12.5 Volume%	12.5 Volume%
Measured Density	6.57 ± 0.05 g/cm^3^	6.58 ± 0.03 g/cm^3^	6.61 ± 0.05 g/cm^3^	6.63 ± 0.04 g/cm^3^	6.63 ± 0.03 g/cm^3^
Relative Density	90.21 ± 0.54 %	90.10 ± 0.77 %	90.26 ± 0.80 %	90.29 ± 0.68 %	90.03 ± 0.77 %
Elastic Modulus (Eb)	95.24 ± 3.16 GPa	104.34 ± 3.77 GPa	113.67 ± 5.83 GPa	122.07 ± 4.93 GPa	131.38 ± 6.66 GPa

## Data Availability

The raw data supporting the conclusions of this article will be made available by the authors on request.

## Abbreviations

The following abbreviations are used in this manuscript:

Eb	Elastic Bond Modulus
K_IC_	Fracture Toughness of wafer
h_p_	Diamond Protrusion Height
CMP	Chemical Mechanical Polishing
BGW	Back-Grinding Wheel
MRR	Material Removal Rate
SSD	Subsurface Damage
WLS	Weighted Least Squares
